# A global analysis of parenchyma tissue fractions in secondary xylem of seed plants

**DOI:** 10.1111/nph.13737

**Published:** 2015-11-09

**Authors:** Hugh Morris, Lenka Plavcová, Patrick Cvecko, Esther Fichtler, Mark A. F. Gillingham, Hugo I. Martínez‐Cabrera, Daniel J. McGlinn, Elisabeth Wheeler, Jingming Zheng, Kasia Ziemińska, Steven Jansen

**Affiliations:** ^1^ Institute of Systematic Botany and EcologyUlm UniversityAlbert‐Einstein‐Allee 11D‐89081UlmGermany; ^2^ Institute of Evolutionary Ecology and Conservation GenomicsUlm UniversityAlbert‐Einstein‐Allee 11D‐89069UlmGermany; ^3^ Institute of Agronomy in the TropicsUniversity of GöttingenGrisebachstrasse 637077GöttingenGermany; ^4^ Département des Sciences BiologiquesUniversité du Québec à MontréalUQÁMCP 8888Succ. Centre Ville MontréalMontréalQCH3C 3P8Canada; ^5^ Department of BiologyCollege of CharlestonCharlestonSC29424USA; ^6^ Department of Forest BiomaterialsNC State University RaleighRaleighNC27695‐8005USA; ^7^ Key Laboratory for Silviculture and Conservation of the Ministry of EducationBeijing Forestry UniversityBeijing100083China; ^8^ Department of Biological SciencesMacquarie UniversitySydneyNSW2109Australia

**Keywords:** angiosperms, axial parenchyma, conifers, growth form, mean annual precipitation, mean annual temperature, ray parenchyma, secondary xylem

## Abstract

- Parenchyma is an important tissue in secondary xylem of seed plants, with functions ranging from storage to defence and with effects on the physical and mechanical properties of wood. Currently, we lack a large‐scale quantitative analysis of ray parenchyma (RP) and axial parenchyma (AP) tissue fractions.
- Here, we use data from the literature on AP and RP fractions to investigate the potential relationships of climate and growth form with total ray and axial parenchyma fractions (RAP).
- We found a 29‐fold variation in RAP fraction, which was more strongly related to temperature than with precipitation. Stem succulents had the highest RAP values (mean ± SD: 70.2 ± 22.0%), followed by lianas (50.1 ± 16.3%), angiosperm trees and shrubs (26.3 ± 12.4%), and conifers (7.6 ± 2.6%). Differences in RAP fraction between temperate and tropical angiosperm trees (21.1 ± 7.9% vs 36.2 ± 13.4%, respectively) are due to differences in the AP fraction, which is typically three times higher in tropical than in temperate trees, but not in RP fraction.
- Our results illustrate that both temperature and growth form are important drivers of RAP fractions. These findings should help pave the way to better understand the various functions of RAP in plants.

## Introduction

**Figure 1:**
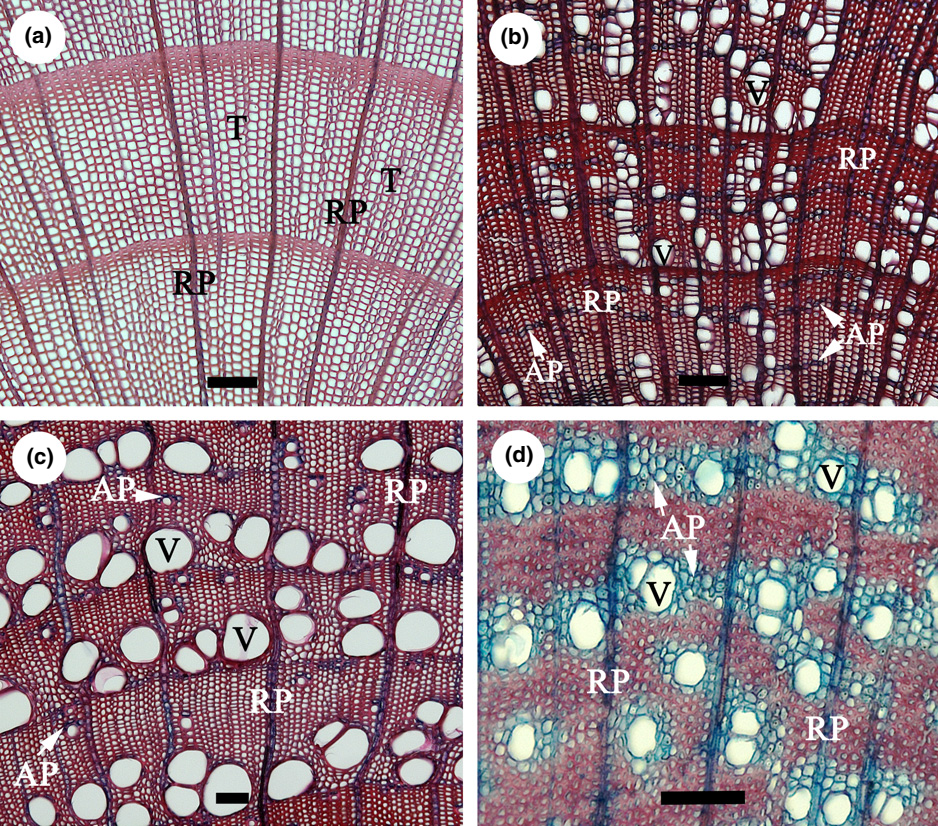
Light microscopy images of transverse sections of conifer and angiosperm stem wood with different parenchyma fractions and spatial distribution, showing a gradient from low to high ray and axial parenchyma percentages. (a) *Picea abies*, a conifer species with no axial parenchyma (AP) present; (b) *Carpinus betulus*, a diffuse‐porous species with narrow bands of AP (white arrows); (c) *Fraxinus excelsior*, a ring‐porous species with both scanty paratracheal AP (white arrows) and marginal bands; and (d) *Crescentia cujeje,* a tropical diffuse‐porous species with aliform and confluent bands of AP (white arrows). The sections were stained with a combination of safranin and alcian blue, resulting in a red colour for strongly lignified cell walls (tracheids (T), vessels (V), and fibres) and a blue to purple colour for both AP and RP (radial parenchyma). All bars, 100 μm.

**Figure 2:**
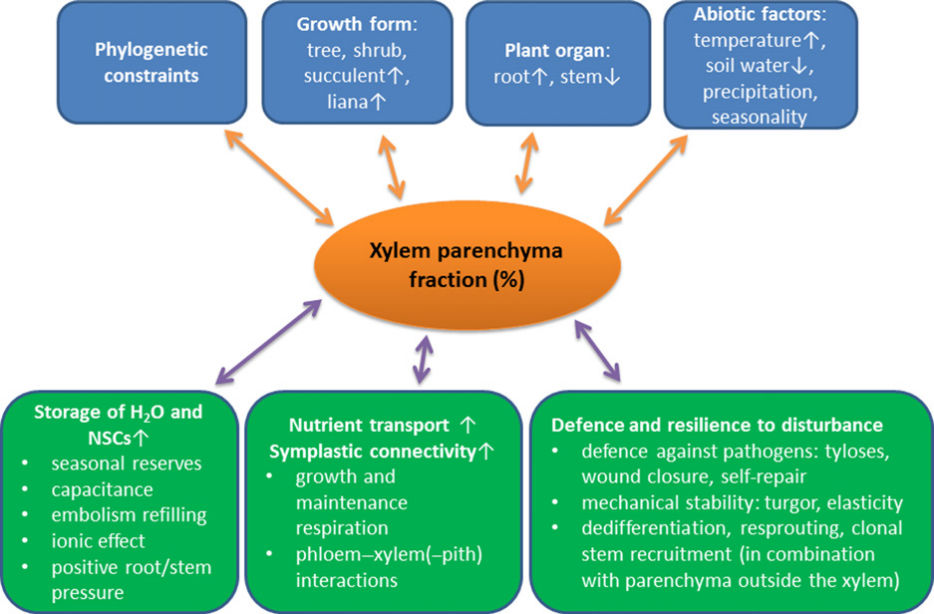
Diagram of variables (in blue) hypothesised to affect xylem parenchyma (including ray and axial parenchyma) tissue fractions in wood and the functions (in green) hypothesised to be related to parenchyma fractions. Arrows pointing upwards or downwards indicate whether or not particular causes may lead to a speculative increase or decrease of parenchyma fraction, or that a functional process is either positively or negatively scaled with parenchyma fraction. Potential variations in parenchyma quantity due to developmental changes and patterns of spatial distribution within the xylem tissue are omitted in this framework. NSC, nonstructural carbohydrates.

Parenchyma tissue in secondary xylem is composed of living cells variable in their morphology and physiology, which usually have thin walls and are rectangular or square in shape. They are produced by fusiform and ray initials of the vascular cambium, which develop into axial parenchyma (AP) strands and ray parenchyma (RP), respectively, and run perpendicular to each other (Fig. 1). Besides the occurrence of so‐called living fibres (Wolkinger, 1970, 1971), ray and axial parenchyma (RAP) tissue represents the bulk of living cells in wood. Parenchyma plays multiple functions, as seen in Fig. 2 (green boxes). These functions range from storage and transport of nonstructural carbohydrates (NSCs) (Hoch *et al*., 2003; Salleo *et al*., 2004; O'Brien *et al*., 2014; Plavcová & Jansen, 2015) to defence against pathogens (Shigo, 1984; Biggs, 1987; Schmitt & Liese, 1993; Deflorio *et al*., 2008), water storage and xylem hydraulic capacitance (Holbrook, 1995; Borchert & Pockman, 2005), storage of mineral inclusions, the transition of functional sapwood to heartwood (Pinto *et al*., 2004; Spicer, 2005; Nawrot *et al*., 2008), and mechanical contributions, particularly by RP (Burgert *et al*., 1999; Burgert & Eckstein, 2001; Reiterer *et al*., 2002). Two additional functions of RAP speculated to be involved in long‐distance water transport are poorly understood, the first being embolism repair (Clearwater & Goldstein, 2005; Salleo *et al*., 2009; Brodersen *et al*., 2010), with the second involving the ion‐mediated enhancement of xylem hydraulic conductance via the release of inorganic compounds such as K^+^ and Ca^2+^ into the transpiration stream (Zwieniecki *et al*., 2001; Jansen *et al*., 2011; Nardini *et al*., 2011; Santiago *et al*., 2013). Radial transport via RAP also needs more exploration. Rays provide means for interactions between phloem and xylem (van Bel, 1990; Spicer & Holbrook, 2007; Hearn *et al*., 2013; Pfautsch *et al*., 2015), as they stretch from the inner bark across the cambium and into the xylem.

RAP shows a large variability in its quantitative and qualitative anatomical characteristics across species (Kribs, 1935, 1937; Barghoorn, 1940, 1941; Normand & Chatelet, 1951; Braun & Wolkinger, 1970; Panshin & de Zeeuw, 1980; Braun, 1984; Koch, 1985). In fact, this variability has been used for wood identification and systematic purposes (IAWA Committee, 1989, 2004; Wagenführ, 2007); however, we lack a thorough understanding of the functional meaning of this variability. Recent advances in image acquisition and analysis techniques have made possible a more accurate and thorough examination of tissue percentages along with the design of studies investigating patterns of variation in total parenchyma fraction (Martínez‐Cabrera *et al*., 2009; Zheng & Martínez‐Cabrera, 2013; Ziemińska *et al*., 2013, 2015).

RAP amount responds to both phylogenetic and environmental factors (Fig. 2). Also, RAP, with the exclusion of rayless species (Carlquist, 2001), is typically higher in angiosperm than in conifer wood for both RP (15–20% compared to 4–8%) and AP (≤ 1% to ≥ 30% compared to ≤ 1%), respectively (Koch, 1985; Spicer, 2014). The level of RAP also depends on growth forms, with a surprisingly high level occurring in woody succulents and lianas (Hearn, 2009). RAP abundance might also change with climate, showing a trend for higher RAP fractions in tropical than in temperate species (Baas, 1982; Wheeler & Baas, 1991). Interestingly, the amount of AP seems to vary considerably between different eco‐regions, both at the intra‐ and interspecific level (Wheeler & Baas, 1991; Segala Alves & Angyalossy‐Alfonso, 2002), but no general trends have been reported for RP as a whole (Fahn *et al*., 1986). As far as we know, there is no published literature on actual comparisons between AP and RP amounts across climatic regions. Different RP compositions may not have any functional advantages across a wide range of climates (Baas, 1982). If this is so, then substantial changes in the overall RP fraction might not be significant, as this paper aims to elucidate. However, RP in *Juniperus thurifera* has been shown to vary in size and abundance annually, suggesting that RP formation in this species is sensitive to interannual changes in precipitation and temperature (Olano *et al*., 2013).

When considering the valuable contributions from earlier studies, major ecological trends in RAP levels remain unclear. The studies that have investigated the association between RAP and climate have either relied on qualitatively classifying species into broadly defined categories based on their RAP abundance (Baas, 1982; Wheeler *et al*., 2007) or were designed as isolated case studies, which were limited in the number of species and sites investigated (Martínez‐Cabrera *et al*., 2009; Olano *et al*., 2013; Ziemińska *et al*., 2015). As far as we are aware, the largest datasets providing detailed information on both climate‐related parameters and RAP levels encompass 61 species growing on eight different sites across South and North America (Martínez‐Cabrera *et al*., 2009) and 69 species collected on three sites along the east coast of Australia (Ziemińska *et al*., 2015). In order to better understand the global variation in RAP levels, which could point to various functional consequences of low vs high RAP fraction, we assembled an extensive dataset that includes many hundreds of species from different climatic zones while using more rigorous quantitative methods to characterise potential trends in RAP fractions. Our main objective was to investigate the association between climate and RAP fraction across a broad range of species and environments. We expect there to be a strong relationship between both temperature and precipitation with RAP, and potentially higher RAP levels in tropical and subtropical species than in temperate species. Also, species that experience drought are hypothesised to show high levels of RAP because of potential correlations of RAP with drought resistance mechanisms such as xylem capacitance and refilling of embolised vessels (Brodersen & McElrone, 2013; Trifilò *et al*., 2014).

We also aimed to elucidate the links between parenchyma fraction and growth form, and to explore differences in RAP amount between organs, namely roots, trunks and branches. We postulate that total RAP fractions are higher in lianas and succulents than in trees due to an increased demand for mechanical elasticity in climbing plants and the importance of water storage in succulents. Across different plant organs, higher RAP and especially RP levels can be expected in roots when compared to stems because of potential differences in storage capacity and mechanical properties (Gasson & Cutler, 1990; Stokke & Manwiller, 1994; Pratt *et al*., 2007). Finally, by way of a large quantitative analysis, we aim to support previous studies (Panshin & de Zeeuw, 1980) that total RAP fraction shows a divergence between angiosperms and conifers. A more detailed phylogenetic investigation of RAP fractions at lower taxonomic levels is beyond the scope of this study and was addressed recently in a range of Chinese species (Zheng & Martínez‐Cabrera, 2013).

## Materials and Methods

### Compilation of the global parenchyma dataset

A total of 1727 records of wood parenchyma percentages for 1439 separate species were obtained from 55 different literature sources (Supporting Information Notes S1). The majority of these records reported values for trunks and branches of trees and shrubs. However, data from small branches of woody climbers and succulents together with data from woody roots were also included. If available, the total fractions of vessels and fibres, representing the other main cell types found in wood, were also collected.

Data on ray parenchyma (RP) and axial parenchyma (AP) were available for 1268 angiosperm records, with 144 and 225 additional records for RP and total ray and axial parenchyma (RAP) (total of 1637 records), respectively. In conifers, only 14 out of a total of 90 records reported values for both AP and RP. This is because AP is sparse in conifers and therefore rarely measured. For this reason, it can be assumed that the total fraction of RP in conifers is equal to the total parenchyma content without introducing substantial bias to the total parenchyma estimate. Orthography and synonymy of species names were checked using the Plant List (v.1.1; http://www.theplantlist.org). In 14 instances we were unable to match the species name reported by the author to any recognised taxon name, and these entries were omitted. Upon pooling together data for trunks and branches reported in the same publication (see the Results section), a core dataset of 1727 entries (1439 species and 127 families) was obtained and used for our analyses. The compiled dataset and corresponding reference list is provided in Table S1, except for the nearly 800 species from Zheng & Martínez‐Cabrera (2013), which are accessible via the TRY Plant Trait Database (https://www.try-db.org/TryWeb/Home.php; Kattge *et al*., 2011).

### Validation of the dataset

High data variability is inherent in large datasets compiled from different literature sources, probably due to different methods used to quantify parenchyma. For example, RAP fractions were based on thin transverse wood sections using light microscopy (Ruelle *et al*., 2006; Martínez‐Cabrera *et al*., 2009; Ziemińska *et al*., 2013, 2015), or on polished wood surfaces using stereomicroscopy (Poorter *et al*., 2010; Fichtler & Worbes, 2012). The relative fraction of parenchyma tissue can be analysed by measuring the entire area covered by the tissue or by estimating this area using a grid overlay (Huber & Prütz, 1938; Smith, 1967). In older books and wood atlases, the method used was not explicitly stated (Panshin & de Zeeuw, 1980; Koch, 1985; Wagenführ, 2007). Another complication is that it can be difficult to distinguish between AP and vasicentric tracheids or thin‐walled living fibres in angiosperms.

As a check of accuracy we compared data taken from the literature to our own data, where we measured RAP fractions for 16 species, out of which 10 species and 14 genera were in our literature‐based dataset. Transverse sections of woody branches 0.5–1 cm in diameter were prepared with a sliding microtome, dehydrated in an ethanol series, stained with a mixture of safranin and alcian blue and mounted in Neo Mount (Merck KGaA, Darmstadt, Germany). Digital images were taken with a stereo zoom microscope (Axio Zoom V16; Zeiss, Germany). A wedge‐shaped region spanning a total area of *c*. 0.5–1.5 mm^2^ from the cambium to the pith was outlined and individual areas taken up by the four principal wood tissues (RP, AP, vessels and imperforate tracheary elements, i.e. fibres and tracheids) within this region were segmented manually in Photoshop with the aid of a graphic tablet (Wacom, Cintiq Companion, model DTH‐W1300; Vancouver, WA, USA). The areas were then measured with ImageJ (Rasband, 1997) and converted to percentage proportions. For 12 temperate species, which were accessible on the Ulm University campus, three small branches from the same individual were measured for each species. For four tropical species, which were available in the glasshouses of the Botanical Garden of Ulm University, one branch could be harvested for measurements, and two radial transects were measured on a transverse section. In total, 16 species (two conifers, nine temperate angiosperms, four tropical angiosperms and one temperate climber) were measured. Our data were then matched to data for 10 species from the compiled dataset. Another comparison was made at the generic level for 14 genera.

### Climate data

In order to investigate correlations between climate and the amount of RAP, AP and RP, we assigned the species into three broadly designated climatic zones: temperate, tropical and subtropical. We used the climatic classification system devised by Köppen (1936), where temperate includes both maritime and continental types, with subtropical ranging from permanent wet to summer dry and winter dry, and tropical including permanent wet, summer dry, winter dry, and monsoonal.

In order to complement the categorical classification, we looked up the spatial coordinates for species in our dataset to serve as proxies for species distribution. We used climate data by way of two different approaches: (1) based on exact locations from the literature, climatic data were obtained for 68 different sampling locations, including 461 different records and 411 species (including both angiosperms and conifers); and (2) where exact locations were not available from the literature, we used the Global Biodiversity Inventory Facility (GBIF), which allowed us to obtain climatic information for 619 species from 612 different locations, covering a wide range of latitudes, longitudes and altitudes (Fig. S1).

For the approach based on exact locations, climatic data for each geographical location were extracted from layers of two major climatic databases using ArcGIS (v.10.0.4.4; ESRI, CA, USA). The layers of the mean annual temperature (MAT, °C) and mean annual precipitation (MAP, mm) were sourced from Bioclim layers based on the World Clim Global Climate Database (Hijmans *et al*., 2005) for the years 1950–2000. The potential evapotranspiration (PET) dataset for each month and the aridity index (AI, which is MAP divided by PET) and mean precipitation of the driest quarter (MPDQ, which is the sum of the average precipitation in the three driest successive months) was taken from the Consortium for Spatial Information (CGIAR CSI). For the GBIF approach, the following criteria were used: (1) the record was not a duplicate according to the spatial coordinates of the sample, (2) we applied a cut‐off at a minimum of 10 records per species for calculating the median location and corresponding climatic computations, and (3) the record was not located within 50 km of the GBIF headquarters in Copenhagen (55.68°N, 12.59°E) to minimise the chance that a record was given a coordinate that corresponded to where the data were housed, but not where the plant was actually collected.

### Statistical analyses

Potential trade‐offs in angiosperm trees between total RAP fraction and the percentage of vessels and fibre (including tracheids) fractions were analysed by plotting these three major xylem tissue fractions on a ternary axis, including a total number of 1302 individual specimens (394 temperate, 428 tropical and 480 subtropical).

We used nonparametric tests due to the lack of data normality. In particular, AP fractions were skewed towards smaller values. The paired‐sample Wilcoxon signed‐rank test was used for evaluating the differences in parenchyma fractions between roots and stems (i.e. any part above soil level) within the same species. A Kruskal–Wallis and a pairwise Wilcoxon test were performed to detect differences in RAP fractions between conifers, angiosperm trees, the two specialized angiosperm growth forms (climbers and succulents), and between angiosperm trees from different climatic zones. Spearman's rank correlation coefficients were calculated to analyse the correlation between the tissue fractions of the three main xylem cell types: RAP, vessels and fibres (including tracheids).

The parenchyma data for which exact locations were known were analysed separately to the data for which only GBIF‐derived climate data was known. We analysed the effect of MAT, MAP and altitude on the proportion of parenchyma using a general additive model (GAM) with a binomial distribution using the mgcv package (Wood, 2006). No further GAM analyses were carried out on PET, AI and MPDQ due to a high co‐linearity between these variables with MAT and MAP (Fig. S2). Each explanatory variable was fitted with a smoother and the maximum effective degrees of freedom (edf, which determines the amount of smoothing) were limited to three partitions. All smooth terms are centred when fitting a GAM in order to ensure model identifiability (Wood, 2006). GAM models were carried out on angiosperms only because the sample size was insufficient for conifers. All statistical analyses were performed using R (R Development Core Team, 2010).

## Results

### Overview of the core dataset

Within the core dataset of 1727 entries, there were 36 records for woody roots and 1691 records for woody stems. The latter can be further subdivided into 1520 records of trunks or branches of angiosperm trees, 89 records of trunks or branches of conifer trees, 32 records of stems from woody climbers and 50 records of stems from woody succulents (see Table 1 for an overview). In general, there was a 29‐fold variation in RAP fractions, with total fractions varying from 3.4% in *Thuja occidentalis* (a coniferous tree) to 99% in *Adenia glauca* (a pachycaul succulent from the Passifloraceae family).

**Table 1:** Data summary of the global xylem parenchyma dataset compiled from the literature with respect to the total number of entries, literature sources, taxa (including angiosperms and gymnosperms) and parenchyma tissue fractions in wood

	RP	AP	RAP
*n* entries	1502	1282	1582
*n* resources	48	38	50
*n* species/genera/families	1265/542/119	1142/518/113	1364/596/123
Mean (%)	17.4	7.2	27.2
Median (%)	16.4	3.3	22.6
Min (%)	2.3	0	3.4
Max (%)	68.4	74	99
cv	45.4	129.8	57.9

RP, ray parenchyma; AP, axial parenchyma; RAP, ray and axial parenchyma; cv, coefficient of variation.

### Validation of the dataset

A close agreement between the literature data and our measurements was found when comparing 10 tree species (*r* ^2^ = 0.571) and 14 genera (*r* ^2^ = 0.920) for total RAP percentages (Fig. S3). The agreement at the genus level was lower for the individual RP and AP data, but still significant (*r* ^2^ = 0.39, *P *<* *0.05, *n *=* *15, and *r* ^2^ = 0.777, *P *<* *0.0001, *n *=* *12 for RP and AP, respectively). However, no significant correlation occurred when comparing the RP and AP fraction data from our measurements with literature data for the same species (*n *=* *14 and 8 for RP and AP, respectively), indicating that there were either potentially important concerns with AP or RP fractions reported in literature for any given species due to varying methodologies, intraspecific differences or interspecific variation. The latter two concerns could be due to developmental age, the organ or sampling position. AP in particular seems to be the most problematic to quantify because identifying AP on transverse sections can be difficult as a consequence of anatomical similarities with thin‐walled living fibres or tracheids (Stokke & Manwiller, 1994; Carlquist, 2014). Therefore, most of our analyses focused on the more robust RAP data, whereas conclusions about the relative contribution of RP and AP should be interpreted with caution.

### Differences between organs, growth forms and angiosperms vs conifers

The differences in parenchyma percentage between roots and stems (including both trunks and branches) were not profound. Slightly higher RP fractions were found in roots than in trunks and branches (paired‐sample Wilcoxon signed‐rank test, *V* = 205, *P *=* *0.04, *n *=* *23), whereas the difference in AP and total RAP was not significant (*V* = 91.5 and 292, *P *>* *0.05, *n *=* *22 and 31, respectively).

Data for both trunks and branches showed no significant difference in RP, AP and RAP fractions (*V* = 237–298.5, *P *>* *0.05, *n *=* *33–34). Therefore, trunks and branches were pooled together and their average was used for further analyses.

**Figure 3:**
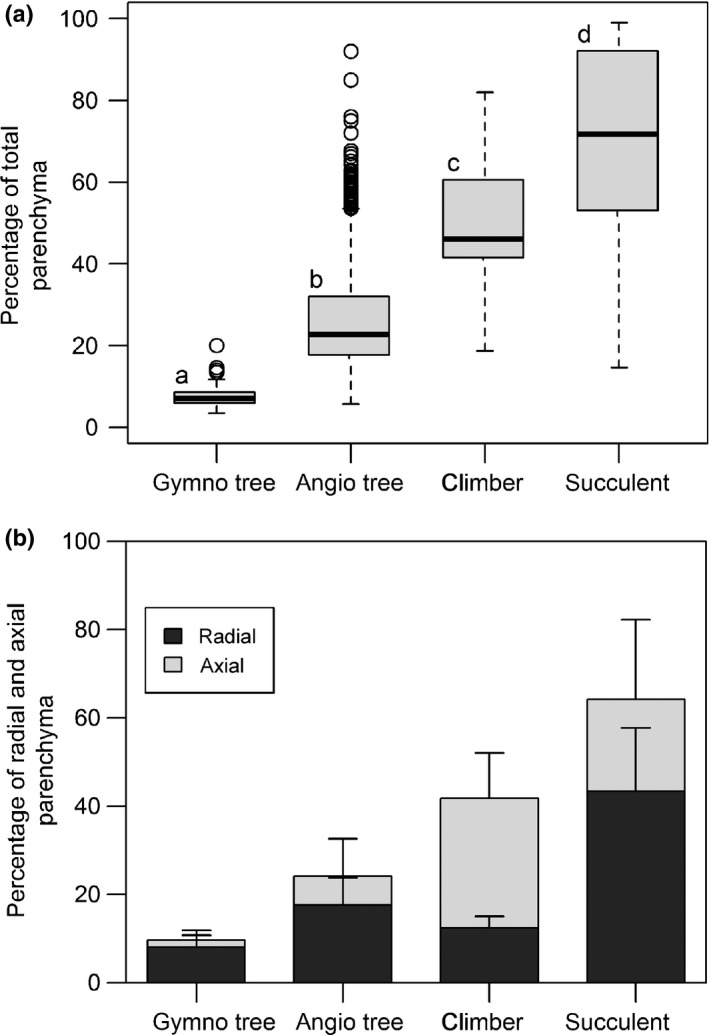
Parenchyma tissue fractions (%) across different woody plant groups and growth forms. Because of inherent differences in wood anatomy between conifer and angiosperm trees, both groups are analysed separately. The lowest parenchyma fractions are found in conifers, followed by angiosperm trees (including some shrubs), climbers and succulents. The percentage of total parenchyma (including axial and ray parenchyma) is shown in (a), with the ray and axial parenchyma contribution for the same groups given in (b). The box plot in (a) shows the median, 25^th^ and 75^th^ percentiles, error bars demarcating 10^th^ and 90^th^ percentiles, and open circles showing outliers. Different letters above boxes indicate statistical significance between groups (Kruskal–Wallis test, *P *≤* *0.05). Bars in (b) represent mean values ± SD. The number of specimens for (a) (with the number of specimens for (b) between brackets) is: *n* gymno tree = 89 (14), *n* angio tree = 1384 (1205), *n* climber = 28 (9), and *n* succulent = 50 (32).

Significant differences in RAP fractions were detected between conifer and angiosperm trees, and between specialised growth forms (stem succulents and lianas), within the angiosperm group, using stem (i.e. trunk or branch) data only (Kruskal–Wallis test, χ^2^ = 118.6, *P *<* *0.001, df = 3; Fig. 3). Stem succulents showed the highest values of RAP (mean ± SD: 70.2 ± 22.0%, *n *=* *50), followed by lianas (50.1 ± 16.3%, *n *=* *28), and angiosperm trees and shrubs (26.3 ± 12.4%, *n *=* *1384), whereas conifers exhibited the lowest fraction of RAP (7.63 ± 2.6%, *n *=* *89, Fig. 3a). In angiosperm trees, there were many entries with rather high RAP, for example 136 entries (118 species) showed total RAP fractions > 50%.

In addition to the total RAP percentage, the contribution of RP and AP was analysed (Fig. 3b), although these data should be interpreted with caution as mentioned above. The information on RP and AP fractions was available for stems of 14 conifer species, 1205 angiosperm tree species, 9 climbers and 32 succulents. Again, conifers showed much lower fractions of both RP and AP (RP, 8.1 ± 2.7%; AP, 1.7 ± 2.2%) than angiosperm trees (RP, 17.7 ± 6.3%; AP, 6.6 ± 8.5%, Fig. 3b). Climbers in our dataset had the highest fraction of AP (29.3 ± 10.2%), whereas RP was relatively low (12.5 ± 2.7%). Succulents showed a high fraction of both RP and AP (RP, 43.5 ± 14.3%, AP, 20.8 ± 18.1%, Fig. 3b).

### Climate and RAP fractions

**Figure 4:**
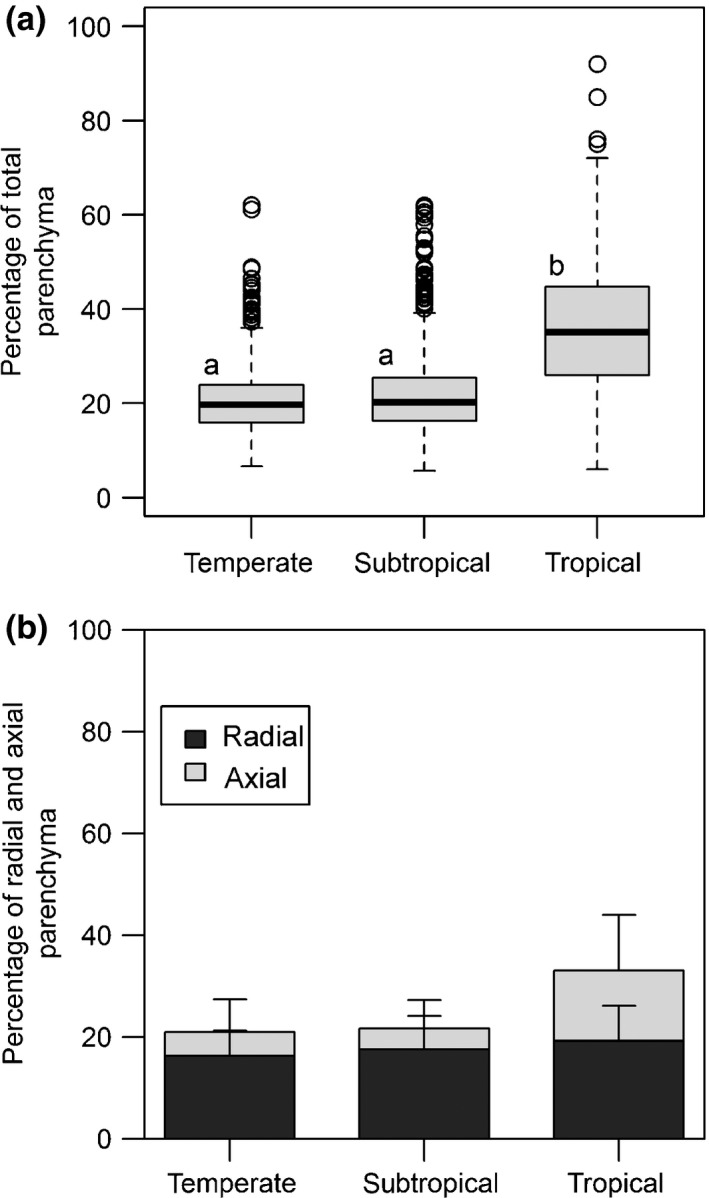
Parenchyma tissue fractions (%) in angiosperm trees classified according to the temperate, subtropical and tropical biome, showing the total parenchyma fraction (a) and the individual contributions of ray and axial parenchyma (b). The box plot in (a) shows the median, 25^th^ and 75^th^ percentiles, error bars demarcating 10^th^ and 90^th^ percentiles, and open circles showing outliers. Different letters above boxes indicate statistical significance for tropical species compared to temperate and subtropical trees (Kruskal–Wallis test, *P *≤* *0.05). Bars in (b) represent mean values ± SD. The number of specimens for (a) (with the number of specimens for (b) between brackets): *n* temperate = 399 (399), *n* tropical = 442 (287), *n* climber = 28 (9), and *n* succulent = 50 (32).

Differences in RAP fractions in angiosperm trees (including some shrubs) growing in various climatic zones were analysed for 399 temperate, 442 tropical and 543 subtropical specimens (Kruskal–Wallis test, χ^2^ = 224.9, *P *<* *0.001, df = 2), with mean values of 21.1% (± 7.9), 22.2% (± 9.3 and 36.2% (± 13.4) for temperate, subtropical and tropical angiosperm specimens, respectively (Fig. 4a). The amount of AP appeared to be the main driver of this difference. The total AP fraction was 13.8% (± 11.0) in tropical trees, whereas it was between 4% and 5% in temperate and subtropical trees (see Fig. 4b). By contrast, average RP fractions spanned a narrow range from 16.4% (± 5) in temperate to 19.4% (± 6.8) in tropical trees.

**Figure 5:**
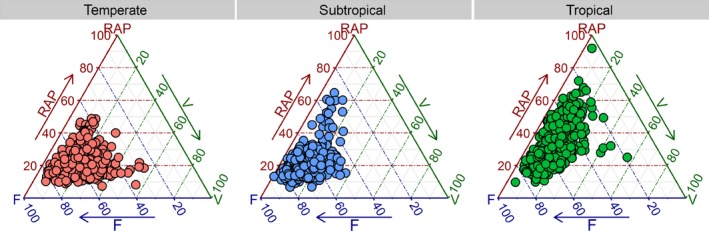
Ternary plot showing the contribution of the three principal tissues in wood: the total amount of ray and axial parenchyma (RAP, %), vessels (V, %), and fibres (including tracheids) (F, %). Each dot represents a specimen. The trade‐off between RAP and F tissue fractions shows a strongly negative correlation for all data and is especially clear in the tropical biome (see Table 2). Specimens are grouped according to three major climatic zones, which follow Köppen (1936).

The ternary axis (Fig. 5) revealed that a higher contribution of RAP occurred mainly at the expense of fibres, particularly in tropical and subtropical trees, whereas total vessel fractions were typically between 5% and 20%, and on average 14.6%. A strongly negative correlation was found between the tissue fractions for RAP and fibres (including tracheids) for all biomes, especially the tropical climate (Spearman's *r *=* *−0.75, *P *<* *0.001 for all biomes; Table 2). Fibre tissue fractions (F) were most negatively correlated with vessel tissue fraction (V) in temperate and subtropical species (*r *=* *−0.66, and −0.59, respectively; *P *<* *0.001), whereas a negative relationship between RAP and V was only weakly significant for temperate and tropical climates (*r *=* *−0.21 and −0.18, respectively; *P *<* *0.001; Table 2). In some temperate trees, the relatively high vessel fractions represented ring‐porous species with narrow growth rings that have a high proportion of early‐wood and, therefore, many wide vessels.

**Table 2:** Spearman's rank correlation coefficients showing the trade‐offs between the tissue fractions of three major xylem cell types, which were analysed for three major biomes (temperate, subtropical, tropical)

Correlation	All biomes	Temperate	Subtropical	Tropical
RAP vs F	−0.752***	−0.480***	−0.688***	−0.856***
RAP vs V	−0.153***	−0.210***	−0.066	−0.176***
F vs V	−0.427***	−0.661***	−0.591***	−0.279***

See Fig. 5 for a visual plot. RAP, ray and axial parenchyma; F, fibres (including tracheids); V, vessels. ***, *P *≤* *0.001.

There was a strong agreement between the climatic data derived from the sampling locations and those derived from the GBIF locations (Fig. S4). There was a clear difference between angiosperms and conifers, as we found only significant correlations between RAP and MAT, and between RAP and MAP for angiosperms. Moreover, due to a low sample number (90 records, 61 species) and limited number of locations, no climatic GAM analyses were performed on conifers.

**Figure 6:**
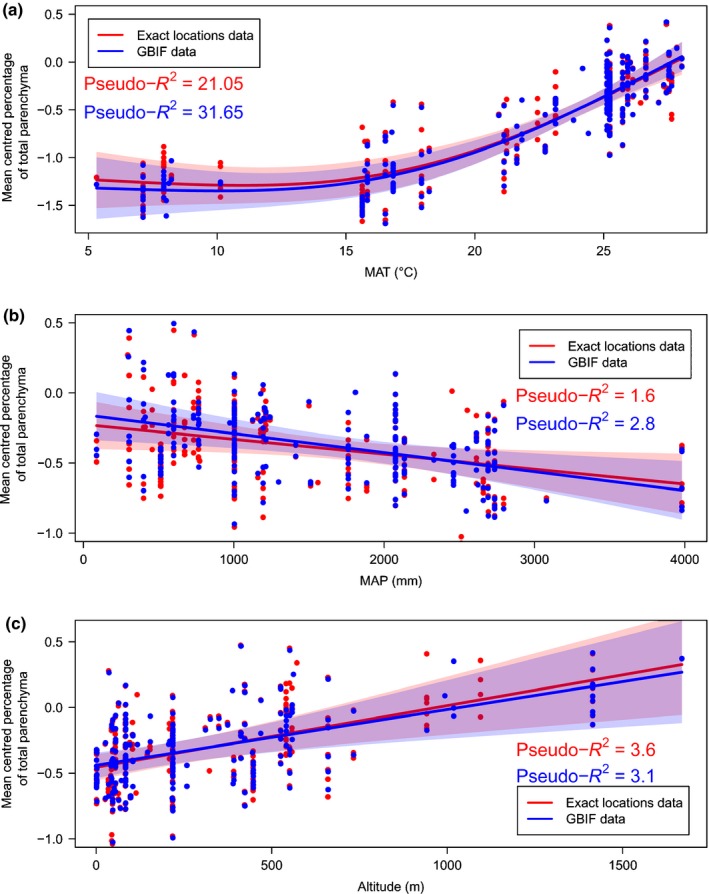
The effect of (a) mean annual temperature (MAT), (b) mean annual precipitation (MAP) and (c) altitude on the proportion of ray and axial parenchyma (RAP) in angiosperm wood based on a general additive model with a binomial distribution for the exact location dataset (red) and the Global Biodiversity Inventory Facility (GBIF)‐derived climate data (blue). Each climate variable was limited to three effective degrees of freedom (e.d.f.). The solid line represents the fitted smoother; 95% confidence intervals are shown as coloured tint areas. Each dot represents a specimen for which the sampling location was reported in literature, or climate data obtained from the WorldClim database. Pseudo‐*R* ^2^ measures the approximate deviance explained by each explanatory variable.

The GAM models showed that MAT was the main driver for RAP in angiosperms (*F*_1.94, 267_ = 37.21, pseudo‐*R* ^2^ = 21.05%, *P*‐value < 0.001; for exact locations, and *F*_1.95, 218_ = 48.22, pseudo‐*R* ^2^ = 31.65%, *P *<* *0.001 for GBIF locations, Fig. 6a). However, the effect of MAT on RAP was nonlinear, with RAP fractions increasing with MAT values in hot environments (> ± 17°C), but not for species in colder environments. When analysing the effect of MAT on AP and RP fractions separately, GAM models revealed significant associations for both when controlling for altitude and precipitation (Fig S5a, S6a; Tables S2, S3).

The relationship between MAP and RAP was also significant, although the pseudo‐*R* ^2^ values were very low compared to those of MAT (Fig. 6b; Tables S2, S3). Although significance was found for the relationship between MAP and RP (Fig. S6b), this was not the case for AP (Fig. S5b; Tables S2, S3). When controlling for MAP and MAT, RAP increased significantly with altitude (Fig. 6c), but these altitudinal trends were nonsignificant or significant for AP and RP, depending on whether the data were based on the exact locations or GBIF (Figs S5c, S6c).

**Figure 7:**
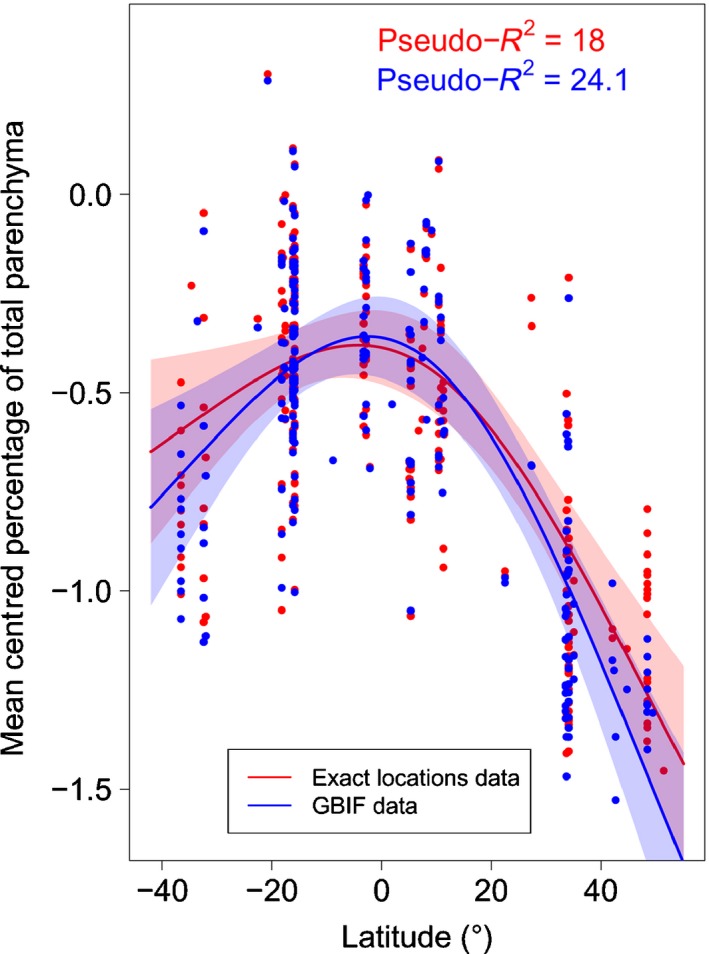
The effect of latitude on the proportion of ray and axial parenchyma (RAP) in angiosperm wood based on a general additive model with a binomial distribution for the exact location dataset (red) and the Global Biodiversity Inventory Facility (GBIF) derived climate data (blue). The latitude variable was limited to three partitions. The solid line represents the fitted smoother; 95% confidence intervals are shown in colour. Each dot represents a specimen for which the sampling location was reported in literature, or climate data obtained from the WorldClim database. Pseudo‐*R* ^2^ measures the approximate deviance explained by latitude.

A separate GAM model between total RAP fraction and temperature also supported a quadratic latitudinal trend (Fig. 7), for both exact locations (*F*_1.95, 267_ = 27.01, pseudo‐*R* ^2^ = 18%, *P *<* *0.001) and species from the GBIF locations (*F*_1.96, 218_ = 31.81, pseudo‐*R* ^2^ = 24.1%, *P *<* *0.001). The latter suggests that RAP increased significantly the closer the sampling location was to the equator for both datasets.

## Discussion

The global ray and axial parenchyma (RAP) dataset compiled demonstrates that RAP tissues show a 29‐fold variation in abundance, which agrees with previous reports across seed plants (Fujiwara *et al*., 1991; Martínez‐Cabrera *et al*., 2009; Zheng & Martínez‐Cabrera, 2013; Ziemińska *et al*., 2013, 2015). A key finding is that RAP amounts in wood, especially axial parenchyma (AP), are driven by temperature (Fig. 6a). Albeit, precipitation also showed a significant relationship with RAP in the general additive model (GAM) models when controlling for altitude and mean annual temperature (MAT), although far less so than temperature (Fig. 6b; Tables S2, S3). Mean annual precipitation (MAP) showed a negative trend in the GAM models, with increasing amounts of RAP towards drier environments, which is what we expected. The temperature effect is also reflected in the latitudinal trends for RAP, especially in the northern hemisphere. However, our analyses did not support the expected difference between subtropical and temperate species (Fig. 4b). Also, no significant difference was found in the RAP tissue fraction between tropical wet environments and tropical, seasonally dry areas (data not shown).

Other drivers of RAP tissue fractions include growth forms. It is clear that lianas and succulents represent two growth forms that show higher fractions of RAP than self‐supporting angiosperm trees. Within the tree and shrub growth form, tropical plants have higher levels of RAP than temperate and subtropical ones (Figs 4, 5), supporting previous studies based on qualitative (Baas, 1982; Segala Alves & Angyalossy‐Alfonso, 2002; Wheeler *et al*., 2007) and quantitative approaches (Martínez‐Cabrera *et al*., 2009). A novel finding is that RAP levels in tropical plants are mainly due to an increase in AP, whereas RP levels remain more conservative in trees across the three major biomes analysed (Fig. 4b). This finding is not as transparent in the GAM models (Figs S4, S5), as both RP and AP are highly associated with temperature. However, where RP amount gradually increases with temperate, AP remains unwavering until *c*. 17°C and then rises sharply and exponentially with temperature. This is the reason AP is so high in the tropics compared to both temperate and subtropical regions (Fig. 4b).

Explaining why some factors may drive the RAP level whereas others have little or no influence requires a more detailed understanding of RAP functions (Fig. 2), especially those related to storage capacity, resistance to drought stress, frost resistance, defence mechanisms and mechanical properties.

### RAP fractions in relation to NSC storage capacity

Storage of nonstructural carbohydrates (NSCs) is arguably one of the most widely accepted functions of RAP in wood. As far as we know, however, the hypothesis that high amounts of RAP correspond to higher storage capacity of NSCs as yet has not been tested in a direct, quantitative way. The assumption that higher amounts of RAP, and therefore a higher NSC capacity, should occur in roots when compared to stems could only partly be confirmed. We found no significant differences between stems and roots in total RAP and AP fractions, but did find higher levels of RP in root wood than in stem wood. As this finding came from a small sample set (*n *=* *21–30), it is premature to make generalisations. A higher fraction of RP in roots than stems has been observed previously (Gasson & Cutler, 1990; Stokke & Manwiller, 1994; Machado *et al*., 2007) and may be associated with a reduced need for mechanical cells such as fibres in addition to increased storage capacity of roots. Aside from RP fractions, different cell dimensions of RP have been reported in roots, especially a general increase in width of the entire ray and the individual ray cells, and a tendency towards more heterocellular rays in roots than in stems (Patel, 1965; Gasson & Cutler, 1990; Denne & Gasson, 2008).

When considering the storage capacity of RAP we would also expect to find higher RAP fractions in plants from temperate seasonal climates. However, we did not find much support for this hypothesis due to lower RAP fractions in trees from temperate compared to tropical biomes. RAP are not the only wood‐tissue storing NSCs; septate or living fibres also do so (Webber, 1936; Yamada *et al*., 2011; Carlquist, 2014).

### RAP fractions and drought stress

It is possible that high RAP fractions in wood benefit plants in dry conditions by conferring high hydraulic capacitance, which could prevent embolism formation, or facilitate embolism refilling (Fig. 6b). Support for a drought related function of RAP is provided by the high levels of RAP in stem succulents. With an average of 70.3% RAP in succulents, it can be speculated that wood parenchyma not only stores a considerable amount of water, but also provides symplastic connections with bark and pith that both serve as important water reservoirs (Borchert & Pockman, 2005; Scholz *et al*., 2007; Hearn, 2009; Hearn *et al*., 2013; Pfautsch *et al*., 2015). However, the large RAP variation in dry and seasonally dry environments suggests that plants have various strategies to survive these conditions, with stem succulents having large parenchyma fractions whereas other species may show comparatively few RAP. It has been suggested that vessel‐associated RAP may be involved in the embolism refilling process by releasing sugars and water into embolised conduits (Bucci *et al*., 2003; Salleo *et al*., 2004; Brodersen *et al*., 2010; Brodersen & McElrone, 2013). Although embolism repair remains controversial and poorly understood, refilling on a daily basis may not occur in conifers, which could be owing to their low RAP fractions (Choat *et al*., 2014).

### RAP fractions and temperature

Temperature is associated with RAP fractions in the secondary xylem, much more so than rainfall. This finding agrees with Moles *et al*. (2014), who found that 15 out of 21 plant traits are more strongly correlated with temperature than with precipitation. The high levels of RAP in tropical plants could be linked to the greater plant diversity in the tropics and the dominance of various families with high RAP levels (e.g. Fabaceae, Moraceae). Alternatively, particular functions associated with RAP (e.g. defence against pathogens, hydraulic capacitance) could be more important in tropical environments than in temperate regions. It is possible that protection against cold, including tolerance to extracellular freezing and freeze dehydration, or freeze avoidance by super‐cooling, is an energy‐demanding process (Quamme, 1991; Neuner, 2014), which could therefore be an important factor in reducing the RAP fraction in woody plants exposed to frost or freezing events. Also, two conifer genera growing in the tropical/subtropical mountains of the southern hemisphere (*Podocarpus* spp. and *Dacrydium* spp.) have higher RAP fractions than conifers from cool temperate regions (Braun, 1984). Similarly, *Pinus canariensis* from the warm islands of Tenerife and La Palma have RAP averages of 14.5%, with AP values accounting for 3% of this (Climent *et al*., 1998).

### RAP fractions as a defence system

RAP fractions might also play a large role in defence against the spread of decay via pathogenic fungi, viruses and bacteria. The presence of RP may prevent the lateral spread of fungi, whereas AP does the same for axial movement (Boddy & Rayner, 1983; Shigo, 1984; Biggs, 1986). Because both RP and AP may accumulate anti‐microbial compounds such as phytoalexins, phenolic compounds and suberin, which all act to inhibit fungal spread (Biggs, 1987), trees with high RAP fractions might be more resistant to brown rot fungi and therefore be better overall compartmentalisers of decay than trees with lower RAP fractions (Schwarze *et al*., 2003).

In agreement with the defence role, the amount of RAP is higher in the sapwood of trees that have recovered from pathogenesis (Tippett & Shigo, 1981; Schmitt & Liese, 1990; Arbellay *et al*., 2010, 2012). Interestingly, another study across seven tree species from the Amazon found that high parenchyma abundance and wide dilating rays were associated with poor compartmentalisation of decay, but this was offset by fast wound closure (Romero & Bolker, 2008). Although in angiosperms, parenchyma cells in contact with vessels can seal off conduits by way of tyloses or gum deposits to avoid the spread of decay (Biggs, 1987; Bonsen & Kučera, 1990; Schmitt & Liese, 1992; Sun *et al*., 2007), defence in gymnosperms mainly lies in the occlusion of tracheids via aspiration of the torus‐margo bordered pits (Fuhr *et al*., 2013), and the production of abundant polyphenolic compounds and traumatic resin ducts in Pinaceae and Cupressaceae (Phillips, 1948; Hudgins *et al*., 2004). Such a strategy is therefore consistent with a lower RAP fraction in conifers. By contrast, a higher fraction of RAP in the tropics might be driven by a greater incidence of biotic stress when compared to temperate environments (Bagchi *et al*., 2014). The evolutionary ‘arms race’ with pathogens and insect herbivores may require the deployment of more RAP or the synthesis of a more diverse suite of secondary compounds by RAP in order to enhance tree defence abilities. However, the trade‐off between RAP and fibre tissue fractions (Fig. 5) may also suggest that high RAP fractions could equally decrease the defence capacity.

### RAP and mechanical properties

Because the amount of RAP occurs mainly at the expense of fibres, one would also expect important effects on wood mechanical properties. It has long been assumed that parenchyma cells often have larger lumina and thinner cell walls than fibres, so high RAP levels should theoretically result in lower wood density together with a reduced stiffness, but retain a higher elasticity. However, rays in *Liquidambar* were found to have a higher specific gravity than surrounding tissues because the ratio of cell wall to lumen was relatively high (Taylor, 1969). Moreover, in a study of 69 angiosperm species it was found that the main driver of the modulus of elasticity (defined as the ratio of tensile stress to tensile strain) was fibre wall fraction rather than RAP fraction (Ziemińska *et al*., 2015). However, that study only looked at species within a small wood density range and the results should be treated with caution.

Several authors have suggested that wide rays, which are common in lianas, allow vessel‐bearing segments to twist without rupturing, which may also explain the occurrence of nonlignified or less lignified ray parenchyma cells in climbing plants (Schenck, 1893; Haberlandt, 1914; Sieber & Kučera, 1980; Gartner, 1991; Putz & Holbrook, 1991). Additional explanations for the high amount of RAP in lianas can be linked with dedifferentiation of parenchyma, allowing rapid recovery from injury, especially after tree fall (Dobbins & Fisher, 1986; Fisher & Ewers, 1991; Busch *et al*., 2010), and their ability to clone readily when detached from the parent plant (Putz, 1984; Yorke *et al*., 2013). A mechanical role of RAP dependent on their turgor pressure has also been suggested in *Adansonia* (Chapotin *et al*., 2006), with RAP in this species occupying as much as 90%.

### General conclusion

The 29‐fold variation in the parenchyma fraction is associated with temperature‐driven differences between tropical, subtropical and temperate woody plants, as well as with different growth forms such as succulents and lianas. The ecological trends discussed suggest ways for further research into how RAP plays a role in woody plant function in the storage of NSCs and water, defence against pathogens and resilience to damage. Various functions of RAP in wood have been suggested and it is generally not clear which function takes precedence in a given situation. Based on the available evidence, this may depend on climate, plant organ, and the potential partitioning in the functional roles of RP and AP (Zheng & Martínez‐Cabrera, 2013). Also, the total RAP percentage does not reflect the spatial, three‐dimensional arrangement of the parenchyma network and its connectivity to other xylem tissues. More research is also needed to test the within tree variation of RAP %, which would involve labour‐intensive studies with careful and well‐planned sampling. Further research based on observational evidence is needed to investigate the role of parenchyma in more detail, such as the spatial patterns of parenchyma networks and, along with this, to test the hypotheses presented in this paper.

## Author contributions

H.M., L.P. and S.J. led the initial data compilation and coordinated the writing. H.M., L.P., S.J., E.F., H.I.M‐C., J.Z. and K.Z. contributed data and ideas. L.P. and M.A.F.G. analysed data. K.Z. and E.W. assisted with writing the final manuscript. H.M., P.C. and D.J.M. extracted GBIF locations and climate data.

## Supporting information

Please note: Wiley Blackwell are not responsible for the content or functionality of any supporting information supplied by the authors. Any queries (other than missing material) should be directed to the *New Phytologist* Central Office.

**Fig. S1 **Distribution map of the species for which parenchyma fraction values were compiled.
**Fig. S2** Poly‐co‐linearity matrix for the parameters analysed in relation to wood anatomy, plant organ, geography and climate.
**Fig. S3 **Comparison of total parenchyma fractions in wood based on our own measurements and literature.
**Fig. S4 **Comparison of mean annual temperature and mean annual precipitation for species for which both sampling locations and GBIF locations were available.
**Fig. S5 **The effect of MAT, MAP and altitude on the proportion of axial parenchyma in angiosperms.
**Fig. S6 **The effect of MAT, MAP and altitude on the proportion of ray parenchyma in angiosperms.
**Table S1 **The Global Wood Parenchyma Database
**Table S2** Summary of statistics for the general additive models (GAM) based on the exact locations dataset
**Table S3** Summary of statistics for the general additive models (GAM) based on the GBIF locations dataset
**Notes S1** Published references from which data were extracted for analyses.
