# Supplementary material for: A global analysis of parenchyma tissue fractions in secondary xylem of seed plants
Source: New Phytol. 2015 Nov 9;209(4):1553–65. doi: 10.1111/nph.13737 (PMC5063116; doi:10.1111/nph.13737)
Supplement: Supplementary file 1 — Fig. S1 Distribution map of the species for which parenchyma fraction values were compiled. Fig. S2 Poly‐co‐linearity matrix for the parameters analysed in relation to wood anatomy, plant organ, geography and climate. Fig. S3 Comparison of total parenchyma fractions in wood based on our own measurements and literature. Fig. S4 Comparison of mean annual temperature and mean annual precipitation for species for which both sampling locations and GBIF locations were available. Fig. S5 The effect of MAT, MAP and altitude on the proportion of axial parenchyma in angiosperms. Fig. S6 The effect of MAT, MAP and altitude on the proportion of ray parenchyma in angiosperms. Table S1 The Global Wood Parenchyma Database Table S2 Summary of statistics for the general additive models (GAM) based on the exact locations dataset Table S3 Summary of statistics for the general additive models (GAM) based on the GBIF locations dataset Notes S1 Published references from which data were extracted for analyses. [file NPH-209-1553-s001.pdf]

New Phytologist

***New Phytologist* Supporting Information**

Article title: **A global analysis of parenchyma tissue fractions in secondary xylem of seed plants**

Authors: Hugh Morris, Lenka Plavcová, Patrick Cvecko, Esther Fichtler, Mark A. F Gillingham, Hugo I. Martínez-Cabrera, Daniel J. McGlinn, Elisabeth Wheeler, Jingming Zheng, Kasia Ziemińska and Steven Jansen

Article acceptance date: 28 September 2015

The following Supporting Information is available for this article:

Fig. S1 Distribution map of the species for which parenchyma fraction values were compiled.

Fig. S2 Poly-co-linearity matrix for the parameters analysed in relation to wood anatomy, plant organ, geography and climate.

Fig. S3 Comparison of total parenchyma fractions in wood based on our own measurements and literature.

Fig. S4 Comparison of mean annual temperature and mean annual precipitation for species for which both sampling locations and GBIF locations were available.

Fig. S5 The effect of MAT, MAP and altitude on the proportion of axial parenchyma in angiosperms.

Fig. S6 The effect of MAT, MAP and altitude on the proportion of ray parenchyma in angiosperms.

Table S1 The Global Wood Parenchyma Database (see separate file).

Table S2 Summary of statistics for the general additive models (GAM) based on the exact locations dataset.

Table S3 Summary of statistics for the general additive models (GAM) based on the GBIF locations dataset.

Notes S1 Published references from which data were extracted for analyses.

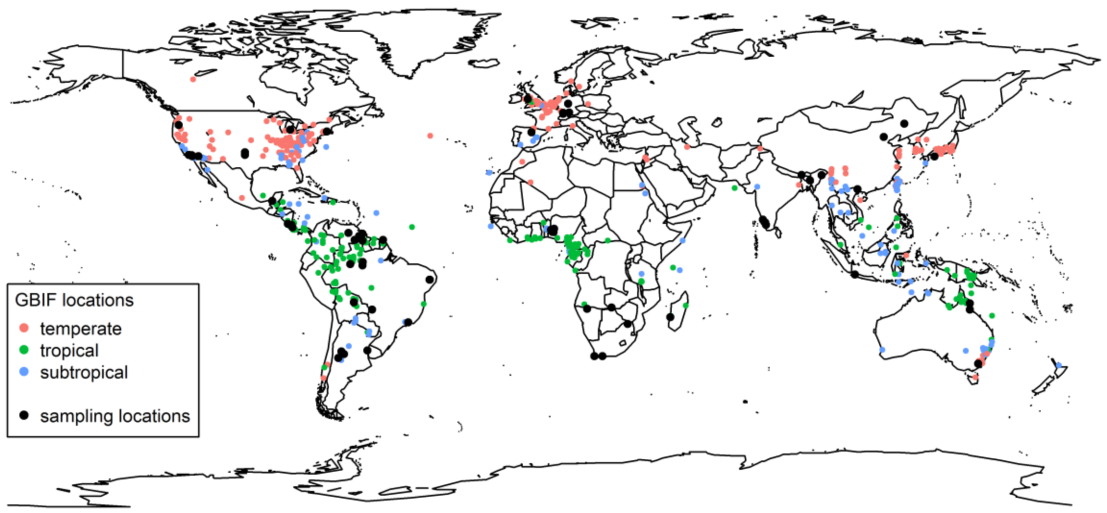

Fig. S1 Distribution map of 612 GBIF locations and 68 exact sampling locations for angiosperm and conifer species for which parenchyma fraction values were compiled in a global xylem parenchyma dataset (see Table S1).

New Phytologist

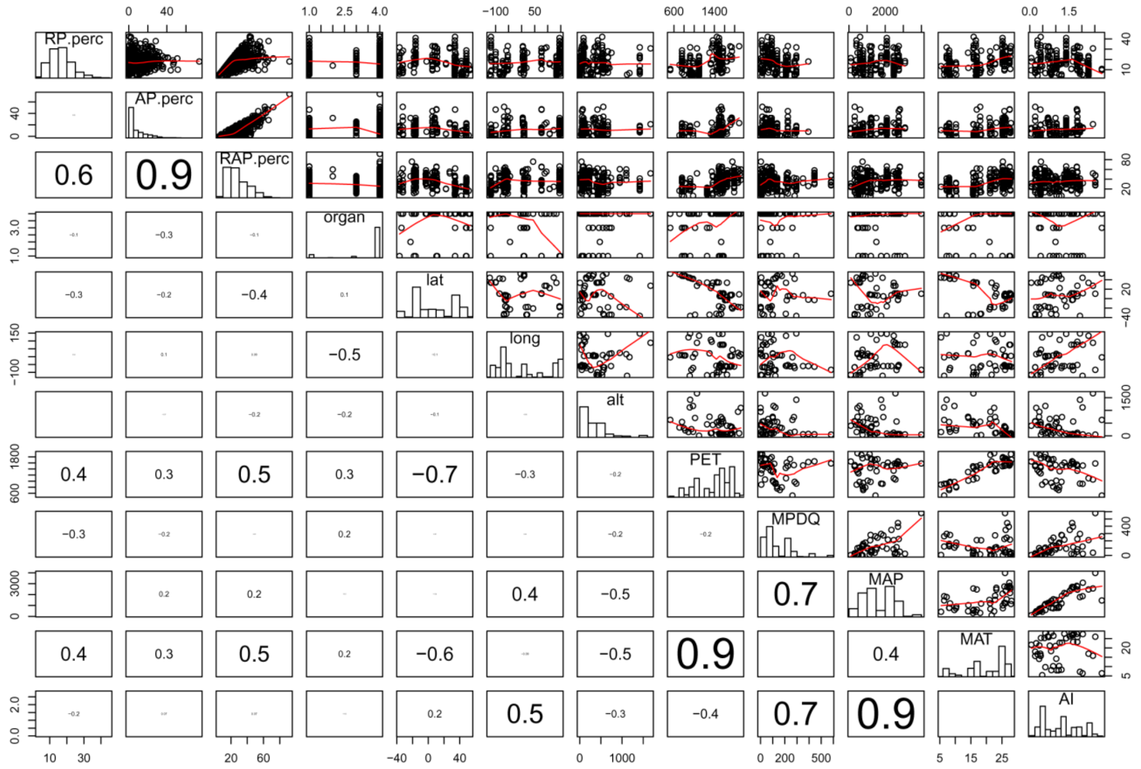

Fig. S2 Poly-co-linearity matrix based on the GBIF dataset for the following parameters: ray parenchyma (RP.perc), axial parenchyma (AP.perc), ray and axial parenchyma (RAP.perc), organ (root, trunk, branch), latitude (lat), longitude (long), altitude (alt), potential evapotranspiration (PET), mean precipitation during the driest quarter (MPDQ), mean annual temperature (MAT), aridity index (AI).

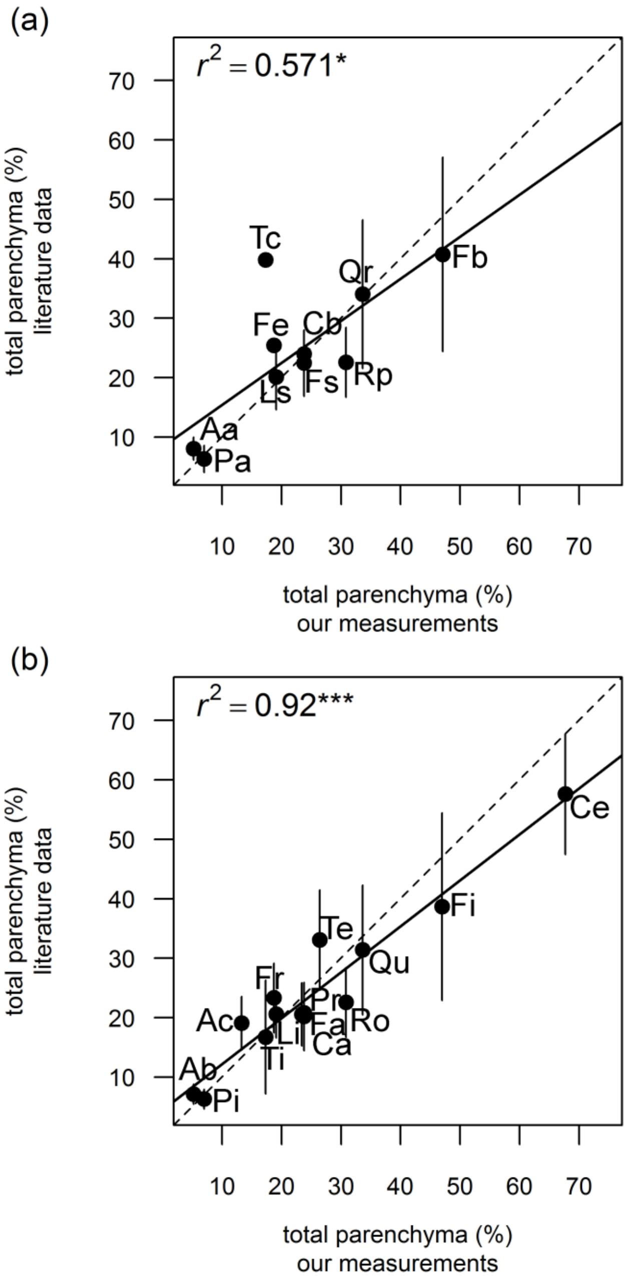

Fig. S3 Comparison of total parenchyma fractions in wood based on our own measurements and data from literature for 10 species (a) and 14 genera (b). The following species were included in Fig. S3(a), with the number of specimens obtained from literature between brackets: Aa, *Abies alba* (3); Cb, *Carpinus betulus* (1); Fs, *Fagus sylvatica* (5); Fb, *Ficus benjamina* (2); Fe, *Fraxinus excelsior* (2); Ls, *Liquidambar styraciflua* (3); Pa, *Picea abies* (4); Qr, *Quercus robur* (3); Rp, *Robinia pseudoacacia* (5); Tc, *Tilia cordata* (1). The genera included in Fig. S3(b) (with

reference to the number of species/specimens from literature) are: Ab, *Abies* (6/9); Ac, *Acer* (14/19); Ca, *Carpinus* (8/8); Cei, *Ceiba* (2/5); Fa, *Fagus* (4/8); Fi, *Ficus* (29/31); Fr, *Fraxinus* (7/12); Li, *Liquidambar* (3/5); *Picea* (5/8); Pr, *Prunus* (9/9); Qu, *Quercus* (39/60); Ro, *Robinia* (1/5); Te, *Terminalia* (9/12); Ti, *Tilia* (9/9). The regression (solid) line and the 1:1 (dashed) line are shown. ***, $P \leq 0.001$; *, $P \leq 0.05$.

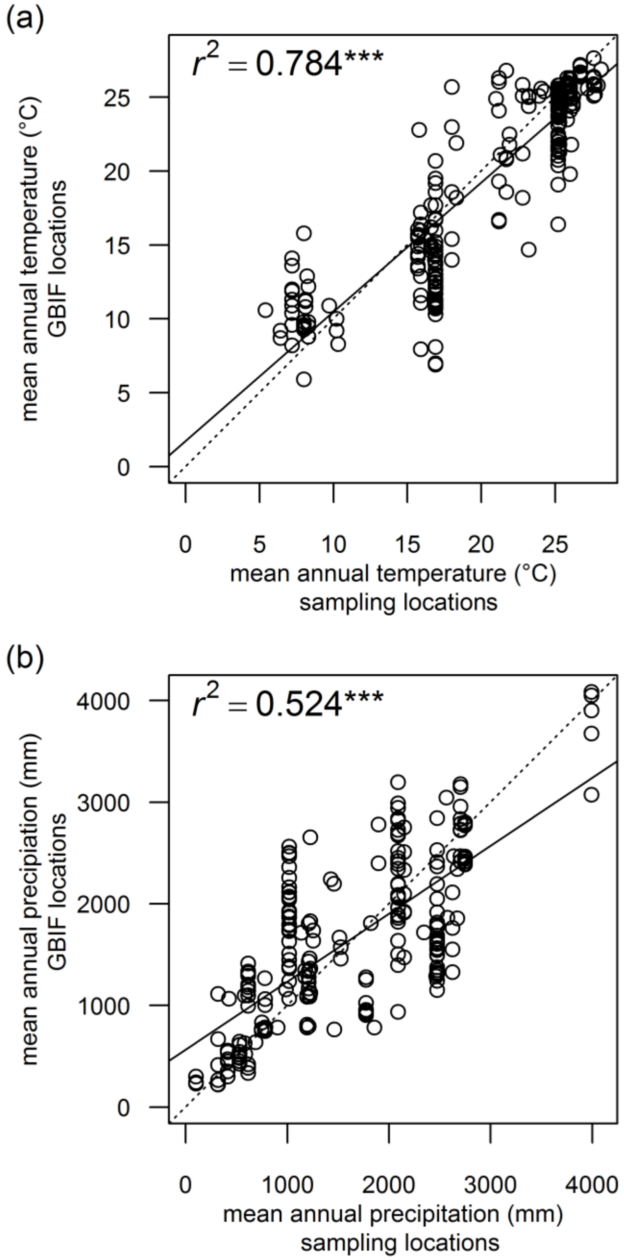

Fig. S4 Comparison of mean annual temperature (a) and mean annual precipitation (b) values for 244 species from the global xylem parenchyma dataset for which both sampling locations and GBIF locations were available. The regression (solid) line and the 1:1 (dashed) line are shown. ***, $P \leq 0.001$.

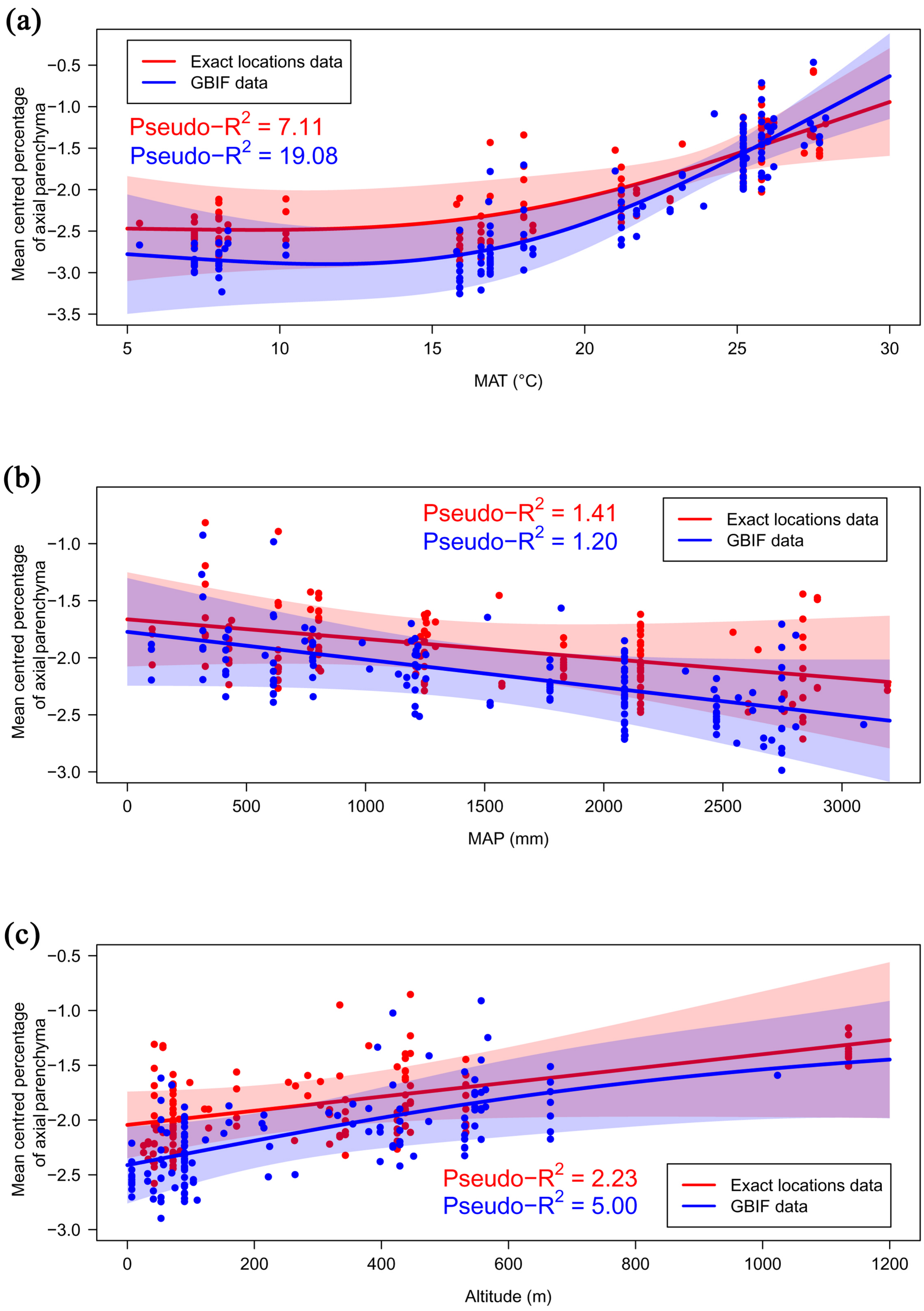
(a)
Exact locations data
GBIF data
Pseudo−R2 = 7.11
Pseudo−R2 = 19.08
Mean centred percentage of axial parenchyma
MAT (°C)
(b)
Pseudo−R2 = 1.41
Pseudo−R2 = 1.20
Exact locations data
GBIF data
Mean centred percentage of axial parenchyma
MAP (mm)
(c)
Pseudo−R2 = 2.23
Pseudo−R2 = 5.00
Exact locations data
GBIF data
Mean centred percentage of axial parenchyma
Altitude (m)

Fig. S5 The effect of MAT (a), MAP (b), and altitude (c) on the proportion of axial parenchyma (AP) in angiosperm wood based on a general additive model (GAM) with a binomial distribution for the exact location dataset (red) and the GBIF derived climate data (blue). Each climate variable was limited to three partitions. The solid line represents the fitted smoother; 95% confidence intervals are shown in colour. Each dot represents a specimen for which the sampling location was reported in literature, or climate data were obtained from the WorldClim database. Pseudo-R^2 measures the approximate deviance explained by each explanatory variable.

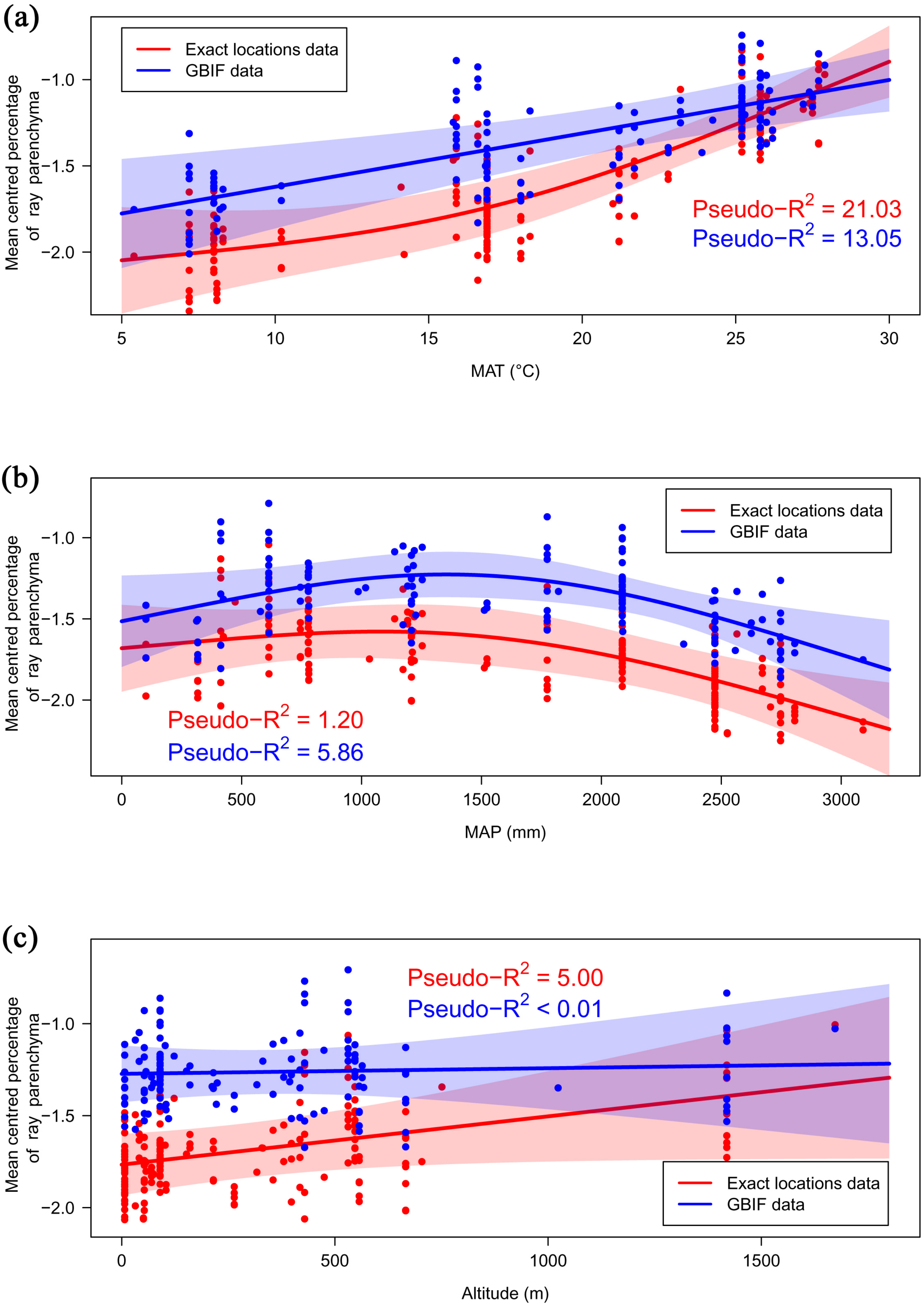
(a)
Exact locations data
GBIF data
Pseudo−R^2 = 21.03
Pseudo−R^2 = 13.05
Mean centred percentage of ray parenchyma
−1.0
−1.5
−2.0
5
10
15
20
25
30
MAT (°C)
(b)
Exact locations data
GBIF data
Pseudo−R^2 = 1.20
Pseudo−R^2 = 5.86
Mean centred percentage of ray parenchyma
−1.0
−1.5
−2.0
0
500
1000
1500
2000
2500
3000
MAP (mm)
(c)
Pseudo−R^2 = 5.00
Pseudo−R^2 < 0.01
Exact locations data
GBIF data
Mean centred percentage of ray parenchyma
−1.0
−1.5
−2.0
0
500
1000
1500
Altitude (m)

Fig. S6 The effect of MAT (a), MAP (b), and altitude (c) on the proportion of ray parenchyma (RP) in angiosperm wood based on a general additive model with a binomial distribution for the exact location dataset (red) and the GBIF derived climate data (blue). Each climate variable was limited to three partitions. The solid line represents the fitted smoother; 95% confidence intervals are shown in colour. Each dot represents a specimen for which the sampling location was reported in literature, or climate data were obtained from the WorldClim database. Pseudo-R^2 measures the approximate deviance explained by each explanatory variable.

Table S1 The Global Wood Parenchyma Database, including 961 records, but excluding Zheng & Martínez-Cabrera (2013). Data were extracted from 55 sources and include species name, plant organ, growth form, and xylem tissue fractions (%) of ray parenchyma, axial parenchyma, and total parenchyma. Specimens were grouped according to three major climatic zones, which follow Köppen (1936).

See separate Excel file.

References

Köppen W. 1936. Das geographische System der Klimate. In: Köppen W, Geiger R, eds. *Handbuch der Klimatologie.* Berlin, Germany: Gebrüder Borntraeger, 1–44.

Zheng J, Martínez-Cabrera HI. 2013. Wood anatomical correlates with theoretical conductivity and wood density across China: evolutionary evidence of the functional differentiation of axial and radial parenchyma. *Annals of Botany* **112**: 927–935.

Table S2 Summary of statistics for the general additive models (GAM) fitted with a binomial distribution for the exact locations dataset. The response variables were RAP, AP and RP. Each explanatory variable was fitted with a smoother with a maximum of three effective degrees of freedom (e.d.f). *P*-values are approximate (Wood, 2006). Pseudo-R^2 measures the approximate deviance explained by each explanatory variable.

**Response variable**	**Explanatory variable**	**e.d.f**	***F*-value**	***P*-value**	**Pseudo-R^2**
**RAP**	MAT	1.945	37.210	< 0.001	21.05%
(*n* = 270)	MAP	1.000	5.219	0.023	1.6%
	Altitude	1.000	12.933	< 0.001	3.6%
**AP**	MAT	1.728	5.108	0.008	7.11%
(*n* = 137)	MAP	1.000	1.471	0.227	1.41%
	Altitude	1.000	2.946	0.088	2.23%
**RP**	MAT	1.000	21.954	< 0.001	21.03%
(*n* = 182)	MAP	1.876	4.964	0.008	1.2%
	Altitude	1.000	3.584	0.060	5.4%

Reference

Wood SN. 2006. *Generalized additive models: an introduction with R.* London, UK: Chapman and Hall.

Table S3 Summary of statistics for the general additive models (GAM) fitted with a binomial distribution for the GBIF locations dataset. The response variables were RAP, AP and RP. Each explanatory variable was fitted with a smoother with a maximum of three effective degrees of freedom (e.d.f). *P*-values are approximate (Wood, 2006). Pseudo-R^2 measures the approximate deviance explained by each explanatory variable.

**Response variable**	**Explanatory variable**	**e.d.f**	***F*-value**	***P*-value**	**Pseudo-R^2**
**RAP**	MAT	1.950	48.234	<0.001	31.65%
(*n* = 221)	MAP	1.000	8.848	0.003	2.8%
	Altitude	1.000	10.433	0.001	3.1%
**AP**	MAT	1.922	14.615	<0.001	19.05%
(*n* = 142)	MAP	1.000	2.955	0.088	1.2%
	Altitude	1.426	14.615	<0.001	5%
**RP**	MAT	1.000	12.988	<0.001	13.05%
(*n* = 142)	MAP	1.876	3.842	0.024	5.86%
	Altitude	1.000	0.044	0.834	<0.01%

Reference

Wood SN. 2006. *Generalized additive models: an introduction with R.* London, UK: Chapman and Hall.

Notes S1 Published references from which data were extracted for analyses.

Bauch J, Dunisch O. 2000. Comparison of growth dynamics and wood characteristics of plantation-grown and primary forest *Carapa guianensis* in Central Amazonia. *International Association of Wood Anatomists Journal* **21:** 321–334.

Bauch J, Quiros L, Noldt G, Schmidt P. 2012. Study on the wood anatomy, annual wood increment and intra-annual growth dynamics of *Podocarpus oleifolius* var. *macrostachyus* from Costa Rica. *Journal of Applied Botany and Food Quality* **80:** 19–24.

Bhat KM, Bhat KV, TK Dhamodaran TK *et al.* 1985. Wood and bark properties of branches of selected tree species growing in Kerala. *KFRI Research Report, Kerala Forest Research Institute* **29:** 1-34.

Bhat KM, Priya PB. 2004. Influence of provenance variation on wood properties of teak from the Western Ghat region in India. *International Association of Wood Anatomists Journal* **25:** 273–282.

Borja de la Rosa A, Machuca R, Fuentes Salinas M, Ayerde Lozada D, Fuentes López M, Quintero Alcantar A. 2010. Caracterización tecnológica de la madera de *Juniperus flaccida* var. Poblana Martínez. *Revista Chapingo. Serie ciencias forestales y del ambiente* **16:** 261–280.

Bravo S. 2010. Anatomical changes induced by fire-damaged cambium in two native tree species of the Chaco region, Argentina. *International Association of Wood Anatomists Journal* **31:** 283–292.

Chapotin SM, Razanameharizaka JH, Holbrook NM. 2006. A biomechanical perspective on the role of large stem volume and high water content in baobab trees (*Adansonia spp.;* Bombacaceae). *American Journal of Botany* **93:** 1251–1264.

Climent J, Gil L, Pardos JA. 1998. Xylem anatomical traits related to resinous heartwood formation in *Pinus canariensis* Sm. *Trees – Structure and Function* **12:** 139–145.

da Silva LB, de Assis Ribeiro dos Santos F, Gasson P, Cutler D. 2011. Estudo comparativo da madeira de *Mimosa ophthalmocentra* Mart. ex Benth e *Mimosa tenuiflora* (Willd.) Poir. (Fabaceae-Mimosoideae) na caatinga nordestina1. *Acta Botanica Brasilica* **25:** 301–314.

das Neves B, Fritz A, Barros CF. 2008. Anatomia do lenhoff de oito espécies de lianas da familia Leguminosae ocorrentes na Floresta Atlântica. *Acta Botanica Brasilica* **22:** 465–480.

Denne P, Gasson P. 2008. Ray structure in root-and stem-wood of *Larix decidua*: implications for root identification and function. *International Association of Wood Anatomists Journal* **29:** 17.

DeSmidt WJ. 1922. Studies of the distribution and volume of the wood rays in slippery elm (*Ulmus fulva* Michx.). *Journal of Forestry* **20:** 352–362.

Dias L, Luizon C, Gasson P. 2012. Anatomical comparison of original and regrowth wood from coppiced and pollarded *Poincianella pyramidalis* trees in the caatinga of Pernambuco, Brazil. *International Association of Wood Anatomists Journal* **33:** 63–72.

Fichtler E, Worbes M. 2012. Wood anatomical variables in tropical trees and their relation to site conditions and individual tree morphology. *International Association of Wood Anatomists Journal* **33:** 119.

French GE. 1923. *The effect of the internal organization of the North American hardwoods upon their more important mechanical properties*. MSc Thesis, New York State College of Forestry, Syracuse, New York, USA.

Fujiwara S. 1992. Anatomy and properties of Japanese hardwoods II. Variation of dimensions of ray cells and their relation to basic density. *International Association of Wood Anatomists Bulletin New Series* **13:** 397–402.

Fujiwara S, Sameshima K, Kuroda K, Takamura N. 1991. Anatomy and properties of Japanese hardwoods I. Variation of fibre dimensions and tissue proportions and their relation to basic density. *International Association of Wood Anatomists Bulletin New Series* **12:** 419–424.

Gartner BL, Baker DC, Spicer R. 2000. Distribution and vitality of xylem rays in relation to tree leaf area in Douglas-fir. *International Association of Wood Anatomists Journal* **21:** 389-401.

Hearn DJ. 2009. Descriptive anatomy and evolutionary patterns of anatomical diversification in *Adenia* (Passifloraceae). *Aliso: A Journal of Systematic and Evolutionary Botany* **27:** 13–38.

Huber B, Prütz G. 1938. Über den Anteil von Fasern, Gefässen und Parenchym am Aufbau verschiedener Hölzer. *European Journal of Wood and Wood Products* **1:** 377–381.

Ishiguri F, Hiraiwa T, Iizuka K, Yokota S, Priadi D, Sumiasri N, Yoshizawa N *et al.* 2009. Radial variation of anatomical characteristics in *Paraserianthes falcataria* planted in Indonesia. *International Association of Wood Anatomists Journal* **30:** 343–352.

Knigge W, Schulz H. 1961. Einfluß der Jahreswitterung 1959 auf Zellartverteilung, Faserlänge und Gefäßweite verschiedener Holzarten. *Holz als Roh-und Werkstoff* **19:** 293–303.

Koch P. 1985. *Utilization of hardwoods growing on southern pine sites*. Asheville, NC, USA: USDA-Forest Service

Lei H, Gartner BL, Milota MR. 1997. Effect of growth rate on the anatomy, specific gravity, and bending properties of wood from 7-year-old red alder *(Alnus rubra)*. *Canadian Journal of Forest Research* **27:** 80–85.

Lei H, Milota MR, Gartner BL. 1996. Between-and within-tree variation in the anatomy and specific gravity of wood in Oregon white oak (*Quercus garryana* Dougl.) *International Association of Wood Anatomists Journal* **17:** 445-461.

Maeglin RR. 1976. Natural variation of tissue proportions and vessel and fiber lengths in mature northern red oak. *Silvae Genetica* **25:** 122-126.

Mauseth J, Plemons-Rodriguez B. 1997. Presence of paratracheal water storage tissue does not alter vessel characters in cactus wood. *American Journal of Botany* **84:** 815–815.

Myer JE. 1922. Ray volumes of the commercial woods of the United States and their significance. *Journal of Forestry* **20:** 337–351.

Ogunkunle ATJ, Oladele FA. 2008. Structural dimensions and paper making potentials of the wood in some Nigerian species of *Ficus* L. (Moraceae). *Advances in Natural & Applied Sciences* **2:** 103-111.

Ogunwusi AA. 2012. Characterization of wood cellular structures of five lesser used wood species growing in Nigeria. *Journal of Natural Sciences Research* **2:** 128-134.

Olano JM, Arzac A, Gárcia-Cervigón AI, Arx G, Rozas V. 2013. New star on the stage: amount of ray parenchyma in tree rings shows a link to climate. *New Phytologist* **198:** 486–495.

Ona T, Ito K, Shibata M, Ootake Y, Ohshima J, Yokota S, Yoshizawa N, Sonoda T. 1999. In situ determination of proportion of cell types in wood by fourier transform raman spectroscopy. *Analytical Biochemistry* **268:** 43–48.

Ona T, Sonoda T, Ito K, Shibata M, Tamai Y, Kojima Y, Ohshima J, Yokota S, Yoshizawa N. 2001. Investigation of relationships between cell and pulp properties in *Eucalyptus* by examination of within-tree property variations. *Wood Science and Technology* **35:** 229–243.

Pande PK, Chauhan L, Singh M. 2005. Wood anatomical variations within the genus *Castanopsis. Journal of Tropical Forest Science* **17:** 366-371.

Panshin AJ, de Zeeuw C. 1980. *Textbook of wood technology. Structure, identification and uses of the commercial woods of the United States and Canada.* New York, USA: McGraw-Hill Book Company.

Petric B, Scukanec V. 1975. Ray tissue percentages in wood of Yugoslavian hardwoods. *International Association of Wood Anatomists Bulletin* **3:** 43-44

Poorter L, McDonald I, Alarcón A, Fichtler E, Licona JC, Peña-Claros M, Sterck F, Villegas Z, Sass-Klaassen U. 2010. The importance of wood traits and hydraulic conductance for the performance and life history strategies of 42 rainforest tree species. *New Phytologist* **185:** 481–492.

Pratt RB, Jacobsen AL, Ewers FW, Davis SD. 2007. Relationships among xylem transport, biomechanics and storage in stems and roots of nine Rhamnaceae species of the California chaparral. *New Phytologist* **174:** 787–798.

Ruelle J, Clair B, Beauchêne J, Prévost MF, Fournier M, *et al*. 2006. Tension wood and opposite wood in 21 tropical rain forest species. 2. Comparison of some anatomical and ultrastructural criteria. *International Association of Wood Anatomists Journal* **27:** 341–376.

Schulz H. 1957. Der Anteil der einzelnen Zellarten an dem Holz der Rotbuche. *Holz als Roh- und Werkstoff* **15:** 113-118.

Spicer R, Holbrook NM. 2007. Parenchyma cell respiration and survival in secondary xylem: does metabolic activity decline with cell age? *Plant, Cell & Environment* **30:** 934–943.

Sreevani P, Rao RV. 2014. Variation in basic density and tissue proportions of *Eucalyptus tereticornis* SM. clones. *Research Journal of Recent Sciences* **4**: 271-274.

Süß H, Müller-Stoll WR. 1972. Zusammenhänge zwischen der Ausbildung einiger Holzmerkmale der Rotbuche (*Fagus sylvatica L.*) und der Jahrringbreite. *Holz als Roh-und Werkstoff* **30:** 342–346.

Taylor FW. 1968. Variations in the size and proportions of wood elements in yellow-poplar trees. *Wood Science and Technology* **2:** 153–165.

Van Geffen KG, Poorter L, Sass-Klaassen U, Van Logtestijn RSP, Cornelissen JHC. 2010. The trait contribution to wood decomposition rates of 15 neotropical tree species. *Ecology* **91:** 3686–3697.

Verdaguer D, Ojeda F. 2002. Root starch storage and allocation patterns in seeder and resprouter seedlings of two Cape *Erica* (Ericaceae) species. *American Journal of Botany* **89**: 1189–1196.

Villagra PE, Roig Juñent FA. 1997. Wood structure of *Prosopis alpataco* and *P. argentina* growing under different edaphic conditions. *International Association of Wood Anatomists Journal* **18:** 37–52.

von Frey-Wyssling A, Aeberli H. 1942. Der Anteil von Fasern, Gefäßen und Parenchym verschiedener Holzarten in Dreiecksdarstellung. *Holz als Roh-und Werkstoff* **5:** 265–268.

Wagenführ R. 2007. *Holzatlas.* Leipzig, Germany: Carl Hanser Verlag.

Woodrum, CL, Ewers FW, Telewski FW. 2003. Hydraulic, biomechanical, and anatomical interactions of xylem from five species of *Acer* (Aceraceae). *American Journal of Botany* **90:** 693–699.

Wu YQ, Hayashi K, Liu Y, Cai Y, Sugimori M. 2006. Relationships of anatomical characteristics versus shrinkage and collapse properties in plantation-grown eucalypt wood from China. *Journal of Wood Science* **52:** 187–194.

Xi, E-H, Zhao G-J. 2011. Research on differentiated xylem cells based on fractal dimension. *BioResources* **6:** 3066-3079.

Yamada Y, Awano T, Fujita M, Takabe K. 2011. Living wood fibers act as large-capacity 'single-use' starch storage in black locust *(Robinia pseudoacacia). Trees – Structure and Function* **25:** 607–616.

Zheng J, Martínez-Cabrera HI. 2013. Wood anatomical correlates with theoretical conductivity and wood density across China: evolutionary evidence of the functional differentiation of axial and radial parenchyma. *Annals of Botany* **112**: 927-935.

Ziemińska K, Wright IJ, Westoby M. 2015. Broad anatomical variation within a narrow wood density range – a study of twig wood across 69 Australian angiosperms. *PLOS ONE* **10**: e0124892.
